# A Decade in Print: The Evolving Academic Benchmark of Cardiology Fellowship Applications

**DOI:** 10.7759/cureus.92810

**Published:** 2025-09-20

**Authors:** Ali Abolhassani, Touré Jones, Arjun N Bhatt, Jackson McClain, Asim Ahmed, Jennifer Davis, Monique Bethel

**Affiliations:** 1 School of Medicine, Medical College of Georgia at Augusta University, Augusta, USA; 2 Department of Libraries, Augusta University, Augusta, USA; 3 Department of Cardiology, Medical College of Georgia at Augusta University, Augusta, USA

**Keywords:** cardiology, education equity, fellowship training, research & development, scholarly output

## Abstract

Background

Research output has become an increasingly important criterion for competitive fellowship applications. While prior studies have quantified this trend in other specialties, the same has not been done for cardiology fellowships.

Objective

The primary objective of this study is to compare pre-fellowship research productivity among incoming cardiology fellows from the Classes of 2017 and 2027, and assess any significant changes in research output between the cohorts. The secondary objective is to evaluate the associations between degree type, institutional mobility, and geographic continuity and research output in the two cohorts.

Methods

This retrospective cohort study included cardiology fellows from two cohorts, 10 years apart, at 10 of the top 25-ranked United States cardiology programs selected from Doximity reputation rankings (Doximity, Inc., San Francisco, California, United States). Biographical data were obtained from publicly available fellowship program websites and Doximity profiles. PubMed was utilized to quantify research output prior to fellowship entry. The primary outcomes were the total number of publications, first-author publications, and cardiology-related (“in-specialty”) publications.

Results

Among 155 fellows, the mean total number of publications increased from 2.67±3.22 in 2017 to 7.18±9.34 in 2027 (p<0.001). First-authorships increased from 1.35±2.25 to 2.54±3.30 (p<0.001), and in-specialty publications rose from 1.79±2.90 to 4.81±8.58 (p<0.001). Logistic regression analysis showed that the 2027 cohort was significantly more likely to have one or more publications (OR 10.19; p < 0.001). Degree type, institutional transitions, and geographic mobility were not significant predictors of the outcome.

Conclusions

Cardiology fellowship applicants in 2027 had markedly higher research output compared to the class of 2017. This trend mirrors patterns in other competitive specialties and indicates a growing emphasis on scholarly productivity in the selection processes. Subgroup patterns should be considered exploratory, given the small sample sizes. These findings also raise questions about equitable access to research opportunities and the potential emergence of barriers to scholarly output among aspiring cardiologists.

## Introduction

The pathway to becoming a board-certified cardiologist has always been a competitive process. The prestige of the field, earning potential, and relatively limited number of fellowship positions contribute to its rank as one of the most competitive internal medicine sub-specialties [1]. Historically, matching into a cardiology fellowship has required top United States Medical Licensing Exam (USMLE) scores, letters of recommendation, and research experience to select the most qualified applicants. The need for applicants to distinguish themselves and improve their chances of acceptance has subsequently led to a prioritization of a specific part of the application: research. It is the aspect of an applicant’s portfolio that demonstrates critical thinking skills and determination, which effectively sets an applicant apart in an increasingly competitive pool of qualified individuals. As a result, applicants across multiple competitive specialties have increased their research output accordingly. The average number of pre-residency publications increased from 2.6 in 2011 to 6.5 in 2018 for neurosurgery, from 3.0 in 2007 to 6.7 in 2014 for orthopedic surgery, and from 1.6 in 2007 to 4.7 in 2018 for dermatology, highlighting this shift in the application process [2-4]. These comparators have extensive longitudinal data that is well-described, unlike internal medicine subspecialties.

Similarly, the cardiology fellowship has also remained competitive, as indicated by its 66% match rate in 2021, compared to 65% in neurosurgery [5,6]. These findings raise an important question about the state of the cardiology application process. With a general trend towards an increase in publications in other competitive specialties, has the trend for cardiology fellowship applicants also changed to meet an elevated research benchmark?

This study's primary aim is to determine whether first-year cardiology fellows in the class of 2027 from 10 of the top 25 cardiology fellowship programs in the United States had significantly different research outputs before matriculation compared to fellows a decade ago. We seek to know whether cardiology fellows in the class of 2027 would have higher research output compared to those in the class of 2017. We hypothesize that research productivity will increase over the decade as applicants become more competitive. To test this hypothesis, we compared cardiology fellows from the class of 2027 with those from the class of 2017. Differences in total publications, first authorships, and in-specialty (cardiology-related) publications were assessed. The secondary aim of this study is to examine how regional and institutional changes influence the evolution of research output as professionals establish deeper roots and connections in a particular location. One study shows that surgeons hired at their home fellowship institution had increased research due to building extensive connections among faculty [7]. We predicted that trainees who remained at one institution might be more academically productive than those who moved during their training. Through the assessment of these factors, this project aims to determine whether there has been a shift in research publication output within the past decade and what factors may affect this change.

## Materials and methods

Study design

A retrospective, observational analysis of cardiology fellows entering training at two distinct periods: the Class of 2017 (Phase 0) and the Class of 2027 (Phase 1). The primary analysis was designed to focus on changes in publication output over a 10-year period in the Postgraduate Year 4 (PGY4) cardiology classes in Phases 0 and 1, including total publication volume, first authorship, and in-specialty publications. The secondary analysis assessed changes in publication output based on Association of American Medical Colleges (AAMC)-defined national regions and institutional changes between the two cohorts [8]. The study was conducted at the Medical College of Georgia at Augusta University.

Program and fellow selection 

Using the Doximity Residency Navigator (Doximity, Inc., San Francisco, California, United States), fellowship programs were filtered by “Internal Medicine,” selected as “Your Specialty,” and “Cardiovascular Disease,” which was chosen as “Intended Fellowship.” Programs were then sorted by “Reputation” [9]. From the top 25 programs, 10 were selected that provided adequate historical data for both Phase 0 and Phase 1 fellows (see Appendices). 

This historical data included the following publicly available information: (1) fellow’s full name; (2) fellow’s degree (MD only, MD/PhD, or International Medical Graduate (IMG)); and (3) fellow’s medical school and residency programs. If the medical school or residency programs were unavailable on the program website, individual fellows were searched on Doximity to identify missing information under “Education & Training.” If these data could not be determined for all cohort members, the following program on Doximity was selected based on reputation.

After applying the inclusion and exclusion criteria, fellows were organized into cohorts, with further subdivisions into MD-only, MD/PhD, and IMG cohorts.

Publication history 

Publication output was determined once fellows were divided into Phase 0 and Phase 1 cohorts with their respective categorical data. Each fellow was searched on PubMed under advanced filter settings. Search settings included “Affiliation” and “Author” names, starting with medical school and then residency. The search format may yield a result similar to "(Doe, John[Author]) AND (Medical Institution[Affiliation])." To ensure common names do not oversaturate the data, the primary data collector would double-check each institution and name match by clicking "expand" next to "Affiliations" within the article on PubMed. This way, it is clear that the same author from the same institution and with the same credentials is included, and common names are left out. Despite this effort, residual misattributions may still be possible. For Phase 0 fellows, the upper limit for the year of publication was set to “2014” to prevent capturing publications after the fellowship began. Once the publications were gathered, each was reviewed to ensure that the names and affiliations matched those of the fellow of interest using the same method of "expanding". In-specialty publications were then determined by examining each publication’s title and scope. For example, keywords related to the field of cardiology or journals specifically focused on cardiology, as determined by a review of the journal's "Aims and Scope" sections, would fall under the "in-specialty" categorization. Finally, total publications, first authorships, in-specialty publications, journal publications, and digital object identifier (DOI) were tabulated into a Google Sheets spreadsheet (Google LLC, Mountain View, California, United States) for data analysis. The impact factor was not considered in this study due to its changing nature over a 10-year period. PubMed-indexed items, including original investigations, reviews, case reports, and letters/editorials, were counted equally in this investigation. This study did not put weight by study design or journal characteristics. Non-PubMed-indexed outputs such as pre-prints and conference abstracts were not included. Poster presentations were not included as a metric as they are inconsistently available and are not reproducible for future investigations.

Regional and institutional history

Following the publication data, each fellow’s medical school, residency, and fellowship programs were organized by AAMC geographic region (Table 1). Changes in the geographic region and institutions that occurred during career transitions were identified and documented. Geographic and institutional mobility were captured as binary transitions (changed vs not changed) without quantifying distance moved, program research intensity, or continuity with mentors. 

**Table 1:** States divided into AAMC regions Data derived from the AAMC website regarding residency application regional names [8]. AAMC: Association of American Medical Colleges

Region	States
New England	Connecticut, Maine, Massachusetts, New Hampshire, Rhode Island, Vermont
Middle Atlantic	New Jersey, New York, Pennsylvania
East North Central	Illinois, Indiana, Michigan, Ohio, Wisconsin
West North Central	Iowa, Kansas, Minnesota, Missouri, Nebraska, North Dakota, South Dakota
South Atlantic	Delaware, District of Columbia, Florida, Georgia, Maryland, North Carolina, Puerto Rico, South Carolina, Virginia, West Virginia
East South Central	Alabama, Kentucky, Mississippi, Tennessee
West South Central	Arkansas, Louisiana, Oklahoma, Texas
Mountain	Arizona, Colorado, Idaho, Montana, Nevada, New Mexico, Utah, Wyoming
Pacific	Alaska, California, Hawaii, Oregon, Washington

Fellow and publication metrics

Fellow metrics included phase (Class of 2017 or Class of 2027), degree (MD only, MD/PhD, IMG), institution change (medical school to residency, residency to fellowship), and geographic region changes based on AAMC regions (medical school to residency, residency to fellowship). Publication metrics included the number of total publications, the number of first-author publications, and the number of cardiology-related (in-specialty) publications.

Statistical analysis

Analyses were executed under Python 3.13.5 with packages pandas 2.3.1, NumPy 2.3.1, SciPy 1.16.0, statsmodels 0.14.5, Matplotlib 3.10.3, seaborn 0.13.2, scikit-posthocs 0.11.4. We calculated descriptive statistics to summarize each fellow’s total publications, first-author publications, in-specialty publications, and changes in institution or geographic region. Continuous variables (e.g., publication counts) were presented as means, medians, and standard deviations (SD), while categorical variables (e.g., degree type, institutional changes) were reported as counts and percentages.

Nonparametric tests (Mann-Whitney U or Kruskal-Wallis) were applied where data were not normally distributed; otherwise, Welch’s t-tests were used. Spearman’s rank correlation coefficients were computed to assess relationships among publication metrics and degree type. We performed chi-square or Fisher’s exact tests for categorical comparisons. Stratified analyses examined differences by degree type (MD, MD/PhD, IMG), and geographic or institutional mobility patterns were compared between cohorts.

Finally, logistic regression models were used to evaluate the likelihood of having ≥1 publication based on phase (Phase 0 vs. Phase 1), degree type, and region/program transitions. All p-values were two-sided, with p<0.05 deemed significant. The Python scripts used in this analysis are available upon request, ensuring reproducibility. No adjustments were made for multiple comparisons. P-values should be viewed cautiously, as Type I error may be inflated because we did not control for family-wise error or false discovery rates

Ethical considerations

The study utilized publicly available data and did not involve direct contact with institutions, fellows, or attending physicians. Therefore, Institutional Review Board approval was not required. Nevertheless, all data were handled in compliance with ethical research standards to protect individual privacy. Because individual-level data were derived from publicly accessible information, there are no privacy concerns. However, the dataset compiled and used for this analysis will not be made publicly available to avoid potential identification of individuals, but will be made available upon reasonable request. For the analysis code, Python scripts used for data analysis are available upon request to ensure reproducibility.

Data validation

Two independent researchers double-checked all data entries. Any discrepancies were resolved through discussion and consensus. Data extraction procedures were standardized to enhance reproducibility and accuracy.

## Results

A total of 72 fellows from the Class of 2017 and 83 fellows from the Class of 2027 were identified, as shown in Table 2. The cohort demographics revealed slight shifts in degree composition, primarily an increase from 4.2% to 10.8% in MD/PhD fellows in the Class of 2027 compared to the Class of 2017. Additionally, there were minor differences in IMG representation, with 12.5% in the Class of 2017 and 8.4% in the Class of 2027. MD-only fellows represented the majority in both cohorts.

**Table 2:** Descriptive comparison of cardiology fellowship cohorts (class of 2017 and 2027) Data was derived from program websites and archives listing current and past fellows (see Appendices) MD: Doctor of Medicine; MD/PhD: Doctor of Medicine and Doctor of Philosophy dual degree; IMG: International Medical Graduate

	Total Fellows, n	MD Only, n (%)	MD/PhD, n (%)	IMG, n (%)
Class of 2017	72	60 (83.3%)	3 (4.2%)	9 (12.5%)
Class of 2027	83	67 (80.7%)	9 (10.8%)	7 (8.4%)

Combined and subgroup publication analysis

The comparative analysis showed noteworthy changes in research output metrics over time (Table 3). Across all fellows, mean total publications rose from 2.67 ± 3.22 in the 2017 class to 7.18 ± 9.34 in 2027 (p<0.001). First-authorships increased from a mean of 1.35 ±2.25 to 2.54 ±3.30 (p<0.001). Similarly, in-specialty publications rose from 1.79 ± 2.90 to 4.81 ± 8.58 (p<0.001). Though the sample size was small, MD/PhD fellows showed an increase in total publications from 0.67 ±1.15 in 2017 to 11.33 ±2.40 in 2027 (p<0.05) and first-author publications from 0.67 ±1.15 in 2017 to 3.67 ±1.73 in 2027 (p<0.05). MD-only fellows demonstrated significant increases in total, first, and in-specialty publications from 2017 to 2027. Total publications for MD-only rose from 2.37 ±2.90 to 6.19 ±9.67 (p<0.001), first-authorships from 1.27 ±2.31 to 2.27 ±3.40 (p<0.001), and in-specialty publications from 1.58 ±2.81 to 4.12 ±8.81 from 2017 to 2027 (p<0.001).

**Table 3:** Comparative Analysis of Research Output Metrics by Cardiology Fellowship Cohorts (Class of 2017 and 2027) Wilcoxon Rank-Sum test was used to compare the two full cohorts with respect to each endpoint. Mann-Whitney U was used to perform comparisons for the subgroup analysis within each degree type. Data were derived from PubMed. Significance is shown by the *p-values, with a significance level of <0.05 MD: Doctor of Medicine; MD/PhD: Doctor of Medicine and Doctor of Philosophy dual degree; IMG: International Medical Graduate

	Class of 2017	Class of 2027	U-value, p-value
Total Fellows	n= 72	n=83	
Total Publications (mean ±SD)	2.67 ± 3.22	7.18 ± 9.34	U=1403, p=<0.001*
First-Authorships (mean ±SD)	1.35 ± 2.25	2.54 ± 3.30	U= 2069, p=<0.001*
In-Specialty Publications (mean ±SD)	1.79 ± 2.90	4.81 ± 8.58	U=1870, p=<0.001*
MD Fellows	n=60	n=67	
Total Publications (mean ±SD)	2.37 ± 2.90	6.19 ± 9.67	U=965, p=<0.001*
First-Authorships (mean ±SD)	1.27 ± 2.31	2.27 ± 3.40	U=1467, p=<0.006*
In-Specialty Publications (mean ±SD)	1.58 ± 2.81	4.12 ± 8.81	U=1224, p=<0.001*
MD/PhD Fellows	n=3	n=9	
Total Publications (mean ±SD)	0.67 ± 1.15	11.33 ± 2.40	U=0, p=0.02*
First-Authorships (mean ±SD)	0.67 ± 1.15	3.67 ± 1.73	U=1, p=0.02*
In-Specialty Publications (mean ±SD)	0.00 ± 0.00	8.44 ± 6.21	U=3, p=0.1
IMG Fellows	n=9	n=7	
Total Publications (mean ±SD)	5.33 ± 4.39	11.29 ± 9.91	U=22, p=0.3
First-Authorships (mean ±SD)	2.11 ± 2.09	3.71 ± 3.64	U=24, p=0.5
In-Specialty Publications (mean ±SD)	3.78 ± 3.23	6.71 ± 8.52	U=31, p=1.0

Among the degree types (MD, MD/PhD, IMG), MDs showed a significant reduction in those with no publications between the two cohorts, decreasing from 18 to 4 (p < 0.05, Table 4). MD/PhD fellows also had a significant reduction of 2 to 0 between Phases (p<0.05). Subgroup analysis is underpowered and should be interpreted as exploratory signals rather than definitive differences. 

**Table 4:** Distribution of non-publishers** by degree and phase Fisher’s exact test was used to assess for significant differences between groups with respect to the number of non-publishers in each. Haldane-Anscombe continuity correction was applied when zero cells were present to enable calculation of finite odds ratios and exact 95% confidence intervals. Odds ratios >1 indicate higher odds of non-publication in the 2017 cohort compared to 2027. Confidence intervals were calculated using exact conditional methods. ** fellows in each graduation year cohort for each degree type (MD, MD/PhD, and IMG) who were found to have zero PubMed-indexed publications. *p < 0.05 considered statistically significant. MD: Doctor of Medicine; MD/PhD: Doctor of Medicine and Doctor of Philosophy dual degree; IMG: International Medical Graduate

Non-publishers Degree	Class of 2017 (Non-published/Total), n	Class of 2027 (Non-published/Total), n	Odds Ratio (95% CI)		Fisher’s p-value
MD	18/60	4/67	6.75 (2.13, 21.35)	p=0.00037*	
MD/PhD	2/3	0/9	31.67 (0.97, 1038.93)	p=0.045*	
IMG	2/9	0/7	5.00 (0.20, 122.74)	p=0.475	

Predictors of publication status

In the multivariable logistic regression model predicting the odds ratio of having one or more publications, only the cohort was a positive predictor. Compared to the 2017 cohort, members of the 2027 cohort were more than ten times as likely to have at least one publication (OR 10.19, CI 95%[3.13, 33.21]). When fellows of different degree statuses were compared in our logistic regression, these differences were rendered insignificant in terms of the probability of producing one publication. Changes in geographic region from medical school to residency and institutional transitions from medical school to residency and residency to fellowship were not predictors of having publications (Table 5). Because multiple predictors were evaluated without multiplicity adjustment, these findings should be viewed as further development of future hypotheses. 

**Table 5:** Logistic regression model for probability of ≥1 publication To identify factors associated with the likelihood of having at least one publication, a multivariate regression model was constructed. The dependent variable was the presence of one or more publications. Independent variables included year cohort, degree type, and geographic transitions in a binary indicator format. Results are shown as odds ratios with the corresponding 95% confidence intervals and p-value. *p-value <0.05 indicates statistical significance. MD: Doctor of Medicine; MD/PhD: Doctor of Medicine and Doctor of Philosophy dual degree; IMG: International Medical Graduate

Predictor	Odds Ratio		Std. Error	z-value	p-value	95% CI
Intercept		2.32	0.596	1.410	0.158	(<1.01, 7.46)
Phase 1 vs Phase 0		10.19	0.603	3.853	<0.001	(3.13, 33.21)
MD/PhD vs MD		0.68	0.947	-0.401	0.688	(<1.01, 4.38)
IMG vs MD		2.11	0.866	0.864	0.388	(<1.01, 11.53)
Region Change (Medical School to Residency)		1.47	0.661	0.580	0.562	(<1.01, 5.37)
Region Change (Residency to Fellowship)		0.68	0.935	-0.418	0.676	(<1.01, 4.23)
Institution Change (Medical School to Residency)		0.35	0.806	-1.296	0.195	(<1.01, 1.71)
Institution Change (Residency to Fellowship)		3.57	0.890	1.430	0.153	(<0.01, 20.47)

## Discussion

Research output, equity, and resource distribution

In this retrospective, observational study of cardiovascular fellows from 2017 to 2027, we noted a significant increase in research productivity. This included all publications, as well as first-author and cardiology-specific publications. This finding is in agreement with findings from other medical subspecialties, including gastroenterology, hematology, endocrinology, and nephrology [10,11]. To our knowledge, this is the first study to examine trends in research output among cardiovascular fellows. These findings highlight a profound evolution in the publication environment for incoming cardiology fellows. 

Our findings indicate that contemporary cardiovascular fellows exhibit greater scholarly productivity than those of a decade ago. An increase in in-specialty publications and first-authorships may represent trainees immersing themselves in focused inquiry, dedicating time to developing their clinical questioning, and assertively taking the lead on projects. The impetus behind this change may reflect a genuine commitment to understanding and improving the scientific foundation of cardiovascular care. However, it is also possible that these accomplishments may be a measure to maintain competitiveness in the current environment. Cullen et al. identified a shift in internal medicine applicants who committed to a cardiovascular fellowship from 2014 to 2024 [1]. These findings included increased scholarly output, cardiovascular medical knowledge, and an earlier declaration of career intent in cardiovascular medicine, possibly reflecting an earlier commitment to the field. This may have implications beyond recruitment for cardiovascular fellows. As the volume of studies swells, concerns arise about the proliferation of less impactful work that clouds the literature with academic “noise” [12]. For this study, we did not collect direct measures of mentorship access, protected research time, funding, or journal quality. Discussion regarding equity and structural barriers is currently speculative, but the conclusions from these data will frame hypotheses for future studies.

Furthermore, the increased need for productivity may inadvertently create barriers to entry for certain applicants. Institutional research support can vary across different residencies. Factors such as the time allocated for research participation during training, the availability of mentors, and institutional funding can vary across programs [13]. Applicants from programs with fewer resources may be at a disadvantage. For applicants with less financial support than others, this could also pose an obstacle to gaining acceptance [14]. Suppose this trend of elevated publication rates continues. Resource-dependent alternatives, such as research years, will likely gain favor but may be out of reach for the average cardiology fellowship applicant. MD/PhDs may be better positioned to thrive amid these evolving expectations, while others may face considerable challenges. The data from this study directly go against this claim, but this is likely due to the limited sample size for MD/PhDs. Therefore, a conclusion cannot be made regarding the MD/PhD deviation from the hypothesized pattern. Given the small sample size for MD/PhD and IMG fellows, we explicitly treat subgroup findings as exploratory and avoid drawing group-level inferences. 

As the ever-evolving benchmarks for fellowship continue to shift toward more extensive research portfolios, competent and motivated applicants who lack these advantages may find themselves at a growing disadvantage. This raises concerns about whether applicants who face professional, logistical, or socioeconomic barriers to accessing research can navigate the intricacies of the academic landscape.

Geographic and institutional mobility

Although the data show substantial increases in publication output, the pattern of geographic region and institutional mobility did not seem to be related to this rise. Theoretically, staying in one region or institution may expand and deepen the roots and networks of medical students, residents, and fellows; however, the logistic regression analysis showed no significance for this hypothesis. These null findings may also reflect limited power and residual confounding rather than a true absence of association. Confounding factors such as personal preferences, obligations, and family situations may contribute to this lack of significance as well. 

Limitations

This project has inherent limitations due to its reliance on publicly available data from top-tier programs, which may not be generalizable to all cardiology fellows. Collecting data from PubMed carries the risk of collecting incorrect data. In attempts to remedy this with affiliation and name matching, there may be a risk of misattribution. Only PubMed-indexed publications were included, as there was difficulty and a lack of standardization in identifying other published works. Additionally, only two cohorts, 10 years apart, do not account for the intervening years, limiting the ability to comment on more gradual trends with confidence. The relatively small representation of MD/PhD and IMG fellows also restricts the strength of these subgroup comparisons, making these results exploratory rather than definitive. As mentioned before, all PubMed-indexed publication types were given equal weight, which may overstate across scholarly contributions. We also performed multiple comparisons without multiplicity correction, increasing the risk of Type I error. Despite making extensive efforts to avoid name ambiguity, misattribution may still be possible as well. Finally, COVID-era publication dynamics were not explicitly taken into account.

Future research

Although this study highlights evolving trends in research output, it also raises new questions that warrant further research. As publication rates increase, we must examine whether this surge enhances or undermines the quality and impact of scholarly contributions. Due to the transition of USMLE Step 1 being scored to pass/fail, more weight will be placed on research involvement. More research development comes with increased financial burdens via resource utilization, which may be limited in less established programs. Research acceptance may also cost thousands of dollars as the median Article Processing Charge (APC) is $2,820, which may add up quickly [15]. There are many open-access publishing journals, but often, APC waivers and discount programs are limited. Further studies should be done to assess the quality, relevance, and scholarly rigor of output from these cohorts. These studies may also include indirect measures of mentorship access, protected research time, funding sources, and journal characteristics. 

## Conclusions

These findings reflect on equity, mentorship, and resource distribution. As the weight of traditional metrics such as USMLE Step 1 scores diminishes, scholarly output has emerged as a cornerstone. With this rise in expectations comes an urgent call to further investigate the existing infrastructure that shapes early academic careers to maintain and cultivate passionate young researchers entering the field of cardiology. It is crucial to address these demands to prevent the exclusion of individuals with fewer resources, less guidance, or limited time. By fostering equitable access to research mentorship and resources, the profession can sustain the growth in scholarly output without sacrificing the diverse talent and nuanced perspectives that enrich patient care and medical discovery. 

## Appendices

Cardiology fellowship programs included for historical data

**Table 6:** The 10 cardiology fellowship programs of the United States included in the study

Program	Website
Mass General Brigham/Massachusetts General Hospital	https://www.massgeneral.org/medicine/internal-medicine
University of California (San Franscisco)	https://medicine.ucsf.edu/residency-programs
New York Presbyterian (Columbia Campus)	https://www.vagelos.columbia.edu/education/residencies-fellowships-and-training/internal-medicine-residency-program
University of California (Los Angeles)	https://www.uclahealth.org/departments/medicine/internal-medicine/im-residency
University of Washington	https://uwmedres.uw.edu
Northwestern University	https://www.medicine.northwestern.edu/education/internal-medicine-residency/index.html
Stanford University	https://med.stanford.edu/medicine/residency.html
University of Chicago	https://imr.bsd.uchicago.edu
Cleveland Clinic Foundation	https://www.clevelandclinic.org/im/res/resident.htm
